# P326: Pharmacovigilance study in côte-d’ivoire on artemisinin derivatives

**DOI:** 10.1186/2047-2994-2-S1-P326

**Published:** 2013-06-20

**Authors:** T Daubrey Potey, M Kamagaté, H Dié-Kacou

**Affiliations:** 1 UFR des Sciences Pharmaceutiques, University Felix Houphoeut Boigny, Abidjan, Côte d'Ivoire

## Objectives

Analyze the various package leaflets on Artemisinin-based combination therapy (ACT) marketed in Côte-d’Ivoire.

## Methods

Comparative study, conducted from June 1 to December 20, 2011 which included all package inserts for all ACT registered and marketed in Côte-d’Ivoire. The inserts’ content was compared to that of European product descriptions; our investigation focused particularly on side effects.

## Results

Regarding artemether-lumefantrine, all leaflets described digestive disorders. As far as endocrinal and metabolic systems are concerned, appetite loss and anorexia were described in 28,5% and 42,8% of leaflets. As far as skin and annexes are concerned, we found: rash (100%), pruritus (90%), slate-gray pigmentation (28%) and redness of the face (14%). Finally, only notices of plasmocid® and coartem® reported biological side effects. As regards the artesunate-amodiaquine, blood effects were described: agranulocytosis (60%), blood dyscrasia and leucopenia (40%), hemolytic anemia (20%). At the gastrointestinal level, were found nauseas, vomiting and diarrhea (80%), hepatitis (60%), fatal hepatitis (20%). On the level of the nervous system was mentioned peripheral neuropathy (80%), the extra-pyramidal syndrome (20%). At the general level, it was asthenia only found in the leaflet of camoquin plus®.

## Conclusion

Information of the laboratories differing from one specialty to another for the same molecule, it would be recommended that these ones harmonize the contents of the leaflets.

## Disclosure of interest

None declared

